# Investigating the direct and indirect effects of a school-based leadership program for primary school students: Rationale and study protocol for the ‘Learning to Lead’ cluster randomised controlled trial

**DOI:** 10.1371/journal.pone.0279661

**Published:** 2023-01-20

**Authors:** Levi Wade, Mark R. Beauchamp, Nicole Nathan, Jordan J. Smith, Angus A. Leahy, Sarah G. Kennedy, James Boyer, Ran Bao, Thierno M. O. Diallo, Josep Vidal-Conti, David R. Lubans

**Affiliations:** 1 Centre for Active Living and Learning, University of Newcastle, Callaghan, New South Wales, Australia; 2 College of Human and Social Futures, School of Education, University of Newcastle, Callaghan, New South Wales, Australia; 3 Active Living Research Program, Hunter Medical Research Institute, New Lambton Heights, New South Wales, Australia; 4 School of Kinesiology, University of British Columbia, Vancouver, British Columbia, Canada; 5 Hunter New England Population Health, Wallsend, New South Wales, Australia; 6 School of Medicine and Public Health, The University of Newcastle, Callaghan, New South Wales, Australia; 7 Hunter Medical Research Institute, New Lambton Heights, New South Wales, Australia; 8 School of Health Sciences, Western Sydney University, Penrith, New South Wales, Australia; 9 New South Wales Department of Education, Sydney, New South Wales, Australia; 10 School of Social Sciences, Western Sydney University, Penrith, New South Wales, Australia; 11 The University of the Balearic Islands, Palma, Spain; 12 Faculty of Sport and Health Sciences, University of Jyväskylä, Jyväskylä, Finland; Public Library of Science, UNITED KINGDOM

## Abstract

**Background:**

Leadership is a valuable skill that can be taught in school, and which may have benefits within and beyond the classroom. Learning to Lead (L2L) is a student-led, primary school-based leadership program whereby older ‘*peer leaders’* deliver a fundamental movement skills (FMS) program to younger ‘*peers’* within their own school.

**Aim:**

The aims of the study are to determine the efficacy of a peer-led FMS intervention on: (i) *peer leaders’* (aged 10 to 12 years) leadership effectiveness (primary outcome), leadership self-efficacy, well-being, and time on-task in the classroom; (ii) *peers’* (aged 8 to 10 years) physical activity levels, actual and perceived FMS competency, cardiorespiratory fitness, muscular power, and executive functioning; and (iii) teachers’ (referred to as ‘*school champions*’) work-related stress and well-being.

**Method:**

L2L will be evaluated using a two-arm parallel group cluster randomised controlled trial. Twenty schools located within a two-hour drive of the University of Newcastle, Australia will be recruited. We will recruit 80 students (40 *peer leaders* and 40 *peers*) from each school (*N* = 1,600). L2L will be implemented in three phases: Phase 1 –*school champions’* training via a professional learning workshop; Phase 2 –*school champions’* delivery of leadership lessons to the *peer leaders*; and Phase 3 –*peer leaders’* delivery of the FMS program to their younger *peers*. The FMS program, consisting of 12 x 30-minute lessons, will be delivered over the course of one school term (10 weeks). Study outcomes will be assessed at baseline (between mid-March to June, Terms 1 and 2), intervention end (mid-August to September, Term 3), and follow-up (November to mid-December, Term 4. This trial was prospectively registered on the Australian New Zealand Clinical Trials Registry (ANZCTR); registration number: ACTRN12621000376842.

## Introduction

‘Leadership’ refers to the behavioural processes through which an individual influences others toward achieving specific goals or objectives [1], and is a valuable life skill that can be developed in the school setting. Providing children with opportunities to develop their leadership skills has been shown to improve their academic outcomes [2], increase their likelihood to take initiative in the classroom [2], and may improve learning autonomy (e.g., studying, completing homework, reviewing lessons etc) [3]. Improving leadership skills at school may also transfer to non-school settings and other life stages (e.g., employment in early adulthood). Surprisingly, there have been very few school-based programs focussed on developing students’ leadership ability. Further, previous peer-led programs have predominantly focused on the benefits for those being led (i.e., *peers*) [4–6], rather than exploring benefits for the *leaders* themselves.

Schools have been identified as key settings for physical activity promotion in children and adolescents [7]. Participating in moderate-to-vigorous intensity physical activity (MVPA) is vital for children’s physical, social, psychological, and cognitive development [8, 9]. Concerningly, less than 20% of Australian children are sufficiently active [10]. Additionally, many children finish primary school without mastering important fundamental movement skills (FMS); e.g., throwing, catching, running, and kicking [10]. While schools are an ideal environment to target children’s physical activity and movement skills, the effects of such interventions are typically modest [11]. The average effect of school-based physical activity interventions on objectively measured MVPA is less than two minutes per day [12]. Further, when interventions are scaled-up, they often experience significant ‘voltage drop’ [13–15]. For example, a 2021 systematic review found that when physical activity interventions are scaled-up, they typically achieve less than 60% of their pre-scale effect size [16]. School-based physical activity interventions can be challenging to implement [17], the problems of which are further compounded when teachers lack the necessary competencies, time, and support to enable effective program delivery [18]. Accordingly, schools require physical activity interventions that are both effective and scalable.

Australian teachers report high levels of work-related stress and burnout [19]. Sources of stress for school teachers include managing student discipline, high workloads, poor working conditions, and lack of support from school administrators [20, 21]. A recent study of Australian teachers (*N* = 960) concluded that improving measures for managing student behaviour was among teachers’ top priorities to improve their own well-being [22]. In line with this, negative student-teacher relationships are associated with lower occupational well-being among teachers [23]. Providing students with opportunities to develop their leadership skills may help to manage their behaviour within and beyond the classroom. This may improve the relationship with their teacher, mitigating teacher stress and improving teacher well-being.

We conducted a pilot study to evaluate a school-based peer leadership program in two primary schools in New South Wales (NSW), Australia (N = 224 students) [24]. The intervention resulted in large improvements in *peer leaders’* effectiveness (*d* = 1.09) and *peers’* FMS competency (*d* = 0.95). Importantly, the program was well received by teachers and students, increasing the likelihood of program dissemination. Although classroom behaviour was not formally measured, teachers anecdotally reported improvements in students’ classroom behaviour following participation in our leadership program. Despite these positive findings, the pilot study involved a quasi-experimental design in two schools and our findings need to be confirmed using a robust cluster randomised controlled trial (RCT).

Previous investigations have demonstrated that peer leadership programs can have a range of benefits for those being led, such as improvements in physical activity [25], mathematical ability [26], and nutrition knowledge [6]. The current project builds upon the findings of our previous pilot study and will examine the effects of a student-led, school-based leadership program known as Learning to Lead (L2L) on students’ behavioural, psychological, and cognitive outcomes. In Australia, students in their last two years of primary school are referred to as Stage 3 students and are typically between the ages of 10 and 12. Henceforth, these students will be referred to as *‘peer leaders’*. Students in the preceding two years of schooling are referred to as Stage 2 students and are typically between the ages of 8 and 10. These students will be referred to as ‘*peers’*. The primary aim is to determine the impact of L2L on *peer leaders’* leadership effectiveness. The secondary aims are to determine the impact of L2L on i) *peer leaders’* self-reported leadership effectiveness, leadership self-efficacy, well-being, and time on-task in the classroom; ii) *peers’* physical activity levels, executive functioning, health-related fitness, and actual and perceived FMS competency; and (iii) teachers’ work-related stress and well-being. We will also examine whether changes in FMS competency mediate changes in executive functioning. Further, this study will be the first to test the hypothesis that improving children’s leadership skills may have ‘spill over’ effects for leaders’ time ‘on-task’ in the classroom and teachers’ work-related stress and well-being.

## Materials and methods

### Research design

**Fig 1:**
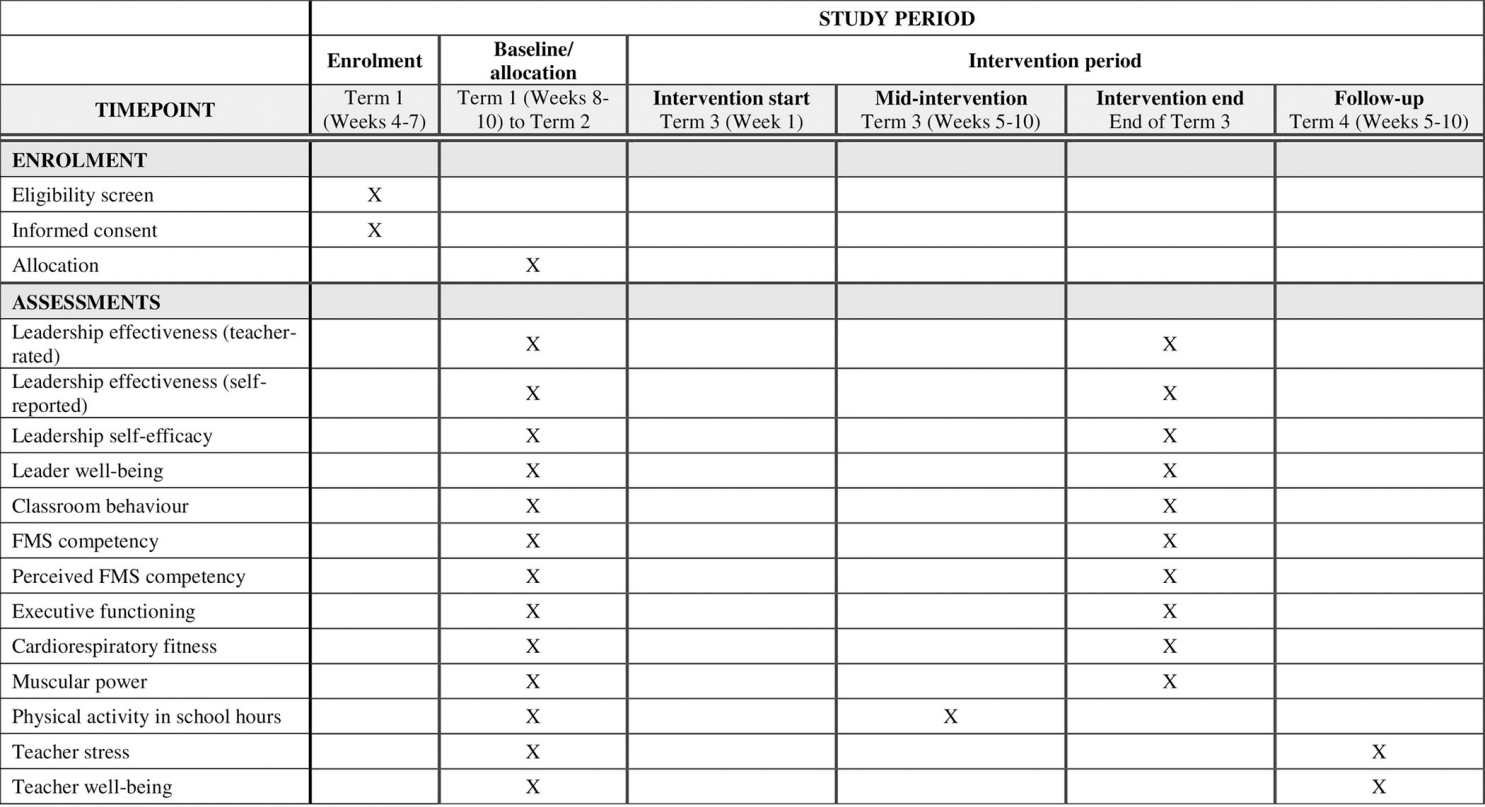
SPIRIT schedule of enrolment, interventions, and assessments. At the time of this paper’s submission, we have begun participant recruitment.

L2L will be evaluated using a two-arm parallel group cluster RCT. Schools will be randomly allocated to a group by an independent researcher via a computer-based random number generator to either an intervention or wait-list control. In Australia, the school year is divided into four terms, each approximately 10 weeks in duration. The study will be conducted across two cohorts (2022 and 2023), with assessments at baseline (between mid-March to June, Terms 1 and 2), intervention end (mid-August to September, Term 3), and follow-up (November to mid-December, Term 4). See Fig 1 for the SPIRIT Schedule Of Enrolment, Interventions, And Assessments. The trial has been prospectively registered with the Australian New Zealand Clinical Trials Registry (registration number: ACTRN12621000376842). The design, conduct and reporting will adhere to CONSORT (Consolidated Standards of Reporting Trials) [27] and TIDieR (Template for Intervention Description and Replication) [28] checklists. The trial has been approved by the University of Newcastle’s ethics committee (reference number: H-2020-0109) and the NSW Department of Education’s State Education Research Applications Process (SERAP; reference number: 2020143).

### Sample size calculation

To ensure 80% power to detect a conservative effect of *d* = 0.50 for leadership effectiveness (adjusted between-group difference of ∼0.4 units with standard deviation of 0.8), we required 128 *peer leaders* for a non-clustered trial (two-tailed, *p*<0.05). However, as recommended by CONSORT [27], we adjusted our power calculation for the clustering of effects at the school level using a correction factor of [1+ (m − 1) × ICC], where m = participants per school and ICC = intraclass correlation coefficient. Assuming an average class size of 20 participating students, two classes per school and an ICC for leadership effectiveness of 0.15, the correction factor is 5.68 [i.e., 1 + (40−1) × 0.15]. The required sample size to achieve 80% power with α levels set at *p* < .05 is 727 (i.e., 128 x 5.68). Operating under the assumption that each school has approximately 60 x Stage 3 students (two classes), with a conservative response rate of 67% and allowing for an expected 10% loss to follow-up, we will aim to recruit at least 40 Stage 3 students from each of 20 schools (*N* = 800). We will also recruit 40 Stage 2 students per school (*N* = 800).

### School recruitment and selection

Government primary schools located within a two-hour drive of the main campus of the University of Newcastle, Australia will serve as the sampling frame. Schools will be eligible if they have at least two Stage 3 classes and are not participating in any other leadership or physical activity programs. Schools will be invited via email and follow-up phone call. In 2022 (Cohort 1), we will randomly select 10 schools from the list of eligible and consenting schools and identify up to 10 replacement schools. Once we have 10 schools, we will pair-match schools according to their size and level of socio-economic disadvantage [29]. The same process will be repeated for Cohort 2 in 2023, until we reach our total sample size of 20 schools.

### Participants

Two Stage 2 (Grades 3 and 4) and two Stage 3 (Grades 5 and 6) classes from consenting schools will be invited to participate. If schools have more than two Stage 2 or Stage 3 classes, the schoolteachers, in consultation with their principal will determine which classes participate. Children from other grades (i.e., Grades 1 and 2) may participate in the FMS program, but they will not be involved in the data collection. Students aged between 7 and 13 are eligible for inclusion. To be included in the research evaluation, parents of students will be required to provide written informed consent, and students will provide assent. Teachers of the Stage 3 classes at consenting schools will be invited to participate in data collection. They must provide written consent to be included in the study.

### Theoretical framework

L2L is guided by transformational leadership theory (not to be confused with transformative leadership) [30], which has a well-established evidence base [31, 32] and is appropriate for educational settings [33]. Research conducted across different settings has demonstrated that transformational leadership is associated with positive outcomes, including higher levels of empowerment [34], motivation [35], and performance [36]. Transformational leaders demonstrate behaviours that empower and inspire others, they transcend their own self-interests and provide others with the confidence to achieve high levels of functioning [30]. Research conducted across different settings has demonstrated that transformational leadership provides a range of positive outcomes for those being led [34–36]. Transformational leadership theory consists of four interrelated behavioural dimensions: (i) *Idealised influence*- fostering trust and respect by role modelling ideal behaviour, (ii) *Inspirational motivation*- displaying optimism, enthusiasm, and having high expectations of others, (iii) *Intellectual stimulation-* encouraging others to consider issues from a different perspective, and (iv) *Individualised consideration*- recognising and supporting others’ physical and psychological needs. Of particular relevance to the current study, it has been demonstrated that: (i) teachers can learn to utilise transformational teaching practices and inspire adolescents to be more physically active [37]; and (ii) Grade 6 students can become transformational leaders in their schools and improve younger children’s fundamental movement skill (FMS) competency [24].

### Intervention description

L2L is a multi-component primary school program co-developed with an advisory group consisting of key stakeholders (education experts, physical activity experts, teachers, and school principals). The program is informed by the tenets of transformational leadership theory [31] and based on previous interventions designed by our research team [24, 38]. Importantly, key stakeholders have recommended the L2L program align with existing school-based leadership opportunities. As such, our research team will work with schools to ensure the tenets of transformational leadership theory are operationalised during the program. The L2L program is guided by the conceptual framework developed by Kelloway and Barling [39] and refined by Beauchamp [31]. This framework involves the following components: (i) explanation of transformational leadership principles, (ii) demonstration of leadership behaviours using real-world examples that are relevant for children, (iii) providing opportunities for children to practice the leadership behaviours, (iv) receiving feedback on the implementation of leadership behaviours, and (v) development of self-regulatory strategies to support sustained implementation. Integral to this framework is the opportunity for *peer leaders* to intellectually engage with the meaning of ‘leadership’ and how their own behaviours can influence others in positive and (if not enacted appropriately) negative ways.

**Fig 2:**
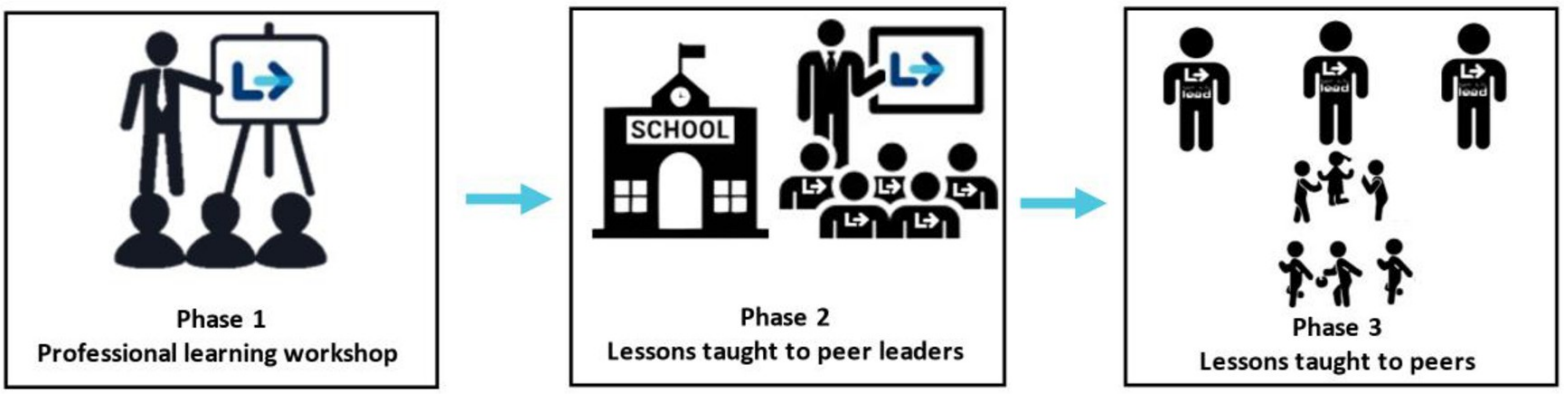
The three phases of learning to lead program.

A description of the intervention components and the evaluation of their implementation is provided in Table 1, and a visual depiction of the three phases of the program are shown in Fig 2.

**Table 1:** Intervention description.

Level	Intervention component	Dose	Description	Implementation evaluation
**School**	External change agent	A minimum of two in-school observations + ongoing support	One external change agent will be allocated to each school. The change agents will come from NSW DoE School Sport Unit or the University of Newcastle. The role of external change agents is to support program implementation. Specifically, they will provide training and on-going support to the school champions. They will also observe two sessions per school over the 10-week study using a structured observation checklist. This information will be provided back to teachers, who will then share this information privately with leaders.	Post-program questionnaire–to gauge acceptability and appropriateness
	Sporting equipment	One pack per Stage 3 class	Each of the participating Stage 3 classes will be provided with a sports equipment pack (i.e., balls, marker cones, cricket bats etc.) to assist in the delivery of the program (valued at ~$1,000 AUD).	Post-program questionnaire–to gauge use and appropriateness of materials
	Posters	Three posters per school	Schools will be provided with L2L posters explaining and promoting the transformational leadership principles.	Post-program questionnaire–to gauge use and appropriateness of materials
**Teacher**	Professional learning Workshop	One full-day workshop	The school champions (teachers of involved Stage 3 classes) will attend a professional learning workshop. The aim of the workshop is to prepare teachers to implement the L2L program in their schools. The workshop includes the following: (i) introduction to the program, (ii) summary of the findings from the pilot study, (iii) importance of physical activity and physical literacy for children, (iv) opportunity for teachers to participate in a L2L session, and (v) opportunity for teachers to make a plan for L2L delivery in their schools.	Workshop evaluation questionnaire
	Curricular materials	One pack per Stage 3 teacher	At the workshop, school champions will be provided with the following curricular materials to deliver the program: L2L unit of work and lesson plans, lesson materials, film clips, and experiential activities and games. All curricular materials will be aligned with the Stage 3 Personal Development, Health and Physical Education curriculum.	Post-program questionnaire–to gauge use and appropriateness of materials
	Learning to Lead–leadership lessons	Six x 40-minute lessons	The school champions will deliver 6 x 40-minute lessons on the program to *peer leaders* in Term 2. The seven lessons will include a mixture of practical activities and information to support leaders to deliver the program. It will address the following four dimensions of leadership: *i) Role modelling*, *ii) Motivating others*, *iii) Considering others*, and *iv) Helping others to think*. Leaders will also be taught the correct movement skill patterns for five object control skills (i.e., catch, underarm throw, overarm throw, bounce, and kick). *Peer leaders* will be trained to provide basic teaching cues, individualised feedback, and opportunities to practice movement skills in a supportive, fun, and encouraging environment.	Post-program questionnaire–to gauge use and appropriateness of materials.
**Leaders**	Lesson plans	One set per leadership group	Intervention schools will be provided with a class set of age-appropriate and purpose-designed laminated “lesson-plans” for leaders to use. *The peer leaders* will be provided with models of the ideal structure of a fundamental movement skill session which includes: (i) *Brief skill introduction*, (ii) *Fast start activity*, (iii) *Skill development* with key teaching points, (iv) *Skill application* in a small-sided game and (v) *Reflection*. While p*eer leaders* will be provided with resources and a recommended session structure, they will have guided autonomy in their delivery of sessions.	Post-program questionnaire–to gauge use and appropriateness of materials
**Leaders and Peers**	Fundamental movement skills (FMS) program	12 lessons	Over the course of a school term, *peer leaders* will deliver 12 FMS lessons to the peers. The sessions will be delivered by 2–3 leaders to a group of 4–6 peers (at a ratio of 1:2). In line with transformational leadership principles, *peer leaders* will remain with the same *peers* for the 10-week duration.	External change agents will collect data on leaders’ adherence to the proposed session delivery. Using a checklist, they will also assess leaders’ adherence to the transformational leadership principles.

### 1) Professional learning workshop for school champions

The *school champions* will attend a full-day professional learning workshop (approximately 6-hours) held at the University of Newcastle. The workshop will be led by members of the research team, and will focus on providing the *school champions* with the knowledge, skills, support, and resources necessary to conduct the L2L program in their schools. The workshop will introduce the program, provide a summary of the findings from the pilot study, explain the how to encourage students to be leaders, highlight the importance of physical activity and physical literacy for children, and provide teachers with the opportunity to participate in a L2L session. During their L2L session, each school champion is given the opportunity to take on the role of *peer* leader (by running part of a session) and as a *peer* (via participation in part of a session). The workshop will conclude by assisting teachers to make an action plan for delivery of L2L in their schools. Here, the *school champions* will be provided with all the curricular materials, equipment, and resources necessary to deliver the program.

### 2) Lessons taught by school champions to *peer leaders*

The *school champions* will deliver 6 x 40-minute lessons to *peer leaders* in Term 2. The six lessons will include a combination of practical activities and information to support *peer leaders* to deliver the FMS program. The information component will address the four aforementioned dimensions of transformational leadership [30], which have been reframed to support *leaders’* understanding as follows: *i) Role modelling*, *ii) Motivating others*, *iii) Considering others*, and *iv) Helping others to think*. Leaders will also be taught the correct movement skill patterns for six object control skills (i.e., catch, underarm throw, overarm throw, bounce, kick, and straight drive [a cricket skill]). *Peer leaders* will be provided with models of the ideal structure of an FMS session which includes: i) *brief skill introduction*, ii) *fast start activity*, iii) s*kill development* with key teaching points, iv) *skill application* in a small-sided game and v) *reflection*. *Peer leaders* will be trained to provide basic teaching cues, individualised feedback, and opportunities to practice movement skills in a supportive, fun, and encouraging environment.

### 3) Lessons taught by *peer leaders* to peers

Over the course of a school term (10 weeks), *peer leaders* will deliver 12 x 30-minute FMS sessions to *peers*. *Peer leaders* will form pairs or groups of three, which will then be paired with a group of 4–6 *peers* (i.e., *peer leader*: *peer* ratio of 1:2) by the *school champions* and the *peers’* classroom teachers. To have the opportunity to apply transformational leadership principles, these *peer leaders* will remain with the same *peers* for the entire 10-week duration. The *peer leaders* will be provided with a class set of age-appropriate and purpose-designed “lesson-plans” to use (see [S1 Fig]). While *peer leaders* will be provided with resources and a recommended session structure, they will have guided autonomy in their delivery of sessions. Based on feedback from our initial pilot study [24], *peer leaders* will be provided with some simple behaviour management techniques to assist managing children’s behaviour, but will also be assisted by supervising classroom teachers in situations where required.

### Control group

To prevent compensatory rivalry and resentful demoralisation, we will use a wait-list control group. Schools allocated to the control group in cohorts 1 and 2 will receive the program in Term 4 of 2022 and 2023, respectively.

### Measures

All assessments will be conducted at the study schools by trained research assistants blinded to intervention assignment. Questionnaires will be completed in exam-like conditions using an online survey with electronic tablets. Standard demographic information will be collected at baseline.

#### Primary outcome (*peer leaders*)

Completed by *school champions* at baseline (Term 1: weeks 8–10 to Term 2), and intervention end (Term 3: weeks 5–10):

*Leadership effectiveness*. *School champions* will be asked to rate *peer leaders’* leadership skills using an adapted version of the Transformational Teaching Questionnaire [40]. The questionnaire was developed to assess the tenets of transformational leadership theory: idealised influence, inspirational motivation, intellectual stimulation, and individual consideration. Using the common stem ‘*The student that I’m rating*…’, the questionnaire includes the following items: ‘behaves as someone that other students can trust’ (idealized influence), ‘is enthusiastic about what other students are capable of achieving’ (inspirational motivation), ‘encourages students to think for themselves’ (intellectual stimulation), and ‘shows that s/he cares about the students s/he is teaching’ (individualised consideration)’. The measure requires respondents to answer four questions for each student, using a five-point scale ranging from ‘0 –*not at all*’ to ‘4 –*frequently’*. The scores of each item are summed to create a composite score. This four-item composite measure of leadership effectiveness was found to have acceptable internal consistency in our pilot (α = 0.89 to 0.92) [24].

#### Secondary outcomes (*peer leaders*)

Completed at baseline (Term 1: weeks 8–10 to Term 2), and intervention end (Term 3: weeks 5–10):

*Self-reported leadership effectiveness*. The *peer leaders* will be asked to complete a self-report of their leadership ability using an adapted version of the Transformational Teaching Questionnaire [40]. Using a five-point scale of ‘0 –*not at all*’ to ‘4 –*frequently’*, *peer leaders* respond to how frequently the following statements are true for them: ‘I show that I care about the students I am teaching’, ‘I am enthusiastic about what other students are capable of achieving’, ‘I encourage students to think for themselves’, and ‘I behave as someone that other students can trust’. The scores of each item are summed to create a composite score.

*Leadership self-efficacy*. *Peer leaders* will be asked to complete an 11-item measure based on standard protocol for assessing leadership self-efficacy [41]. *Peer leaders* will be asked to rate their confidence to perform the key leadership behaviours being targeted in the intervention. Responses to items are provided on a 0–100 scale (with 10-point increments) anchored by 0% (“Not at all confident”), 50% (“Somewhat confident”), and 100% (“Completely confident”). The items were adapted from a previous questionnaire [42], with each question prefaced with “If you really wanted to, how confident are you that you can…”, with follow-up items including “…be a role model to other students”, and “…teach physical activity skills to other students”.

*Well-being*. The Short Warwick Edinburgh Mental Well-Being Scale (SWEMWBS) will be used to assess *peer leader* well-being [43]. The SWEMWBS requires one to indicate how much they agree or disagree with a series of seven statements regarding their well-being overt the past two weeks. The responses to each statement are on a five-point scale, ranging from ‘Disagree a lot’ to ‘Agree a lot’. This scale has been shown to be an appropriate measure of mental well-being in young people [44].

*Time-on*-*task in the classroom*. Classroom observations will be conducted by trained research assistants using established methods [45]. To increase standardisation across schools, where possible the observations will take place during Mathematics or English lessons at the same time of day. During each 30-minute observation period (starting 5 minutes after students enter the classroom), research assistants will assess the on-task and off-task behaviour of six randomly selected students (5 minutes per student). For each lesson, two observers will randomly select 6 boys and 6 girls (i.e., 12 students in total) and the order in which they are observed (teachers and students will not know who is being observed). The same students will be assessed at the end of the intervention. Observers will listen to an audio file via headphones, which will inform them when to observe and record (in 15 second intervals). After each interval, the observers will record the student’s behaviour by recording an appropriate code (i.e., actively engaged, passively engaged, off-task motor, off-task verbal, or off-task passive) using an observation recording sheet. Time spent on- and off-task during the lesson will be expressed as a percentage of the 30-minute classroom observation.

#### Secondary outcomes (*peers*)

Actual and perceived object control FMS competency, and health-related fitness will be assessed at baseline (Term 1: weeks 8–10 to Term 2), and intervention end (Term 3: weeks 5–10). Physical activity will be measured at baseline and mid-intervention:

*Actual FMS competency*. Will be assessed using a subset of object control skills (i.e., overarm throw, kick, and catch) from the Test of Gross Motor Development 3 [46]. Children will be filmed performing three trials (inclusive of one practice trial) of each of the three skills. These skills were selected due to their transferability into a variety of different sports that are popular among Australian children. Research assistants will assess the videos according to the performance criteria. Each skill component will be scored a “1” if observable and performed correctly or “0” if performed incorrectly. This procedure will be completed for each of the two main trials, and trial scores will be summed to calculate a total score for each skill. An overall object control movement skill score will be calculated by summing the skill scores.

*Perceived FMS competency*. Perceived competence in the same three fundamental movement skills described above will be assessed using the Pictorial Scale of Perceived Movement Skill Competence, which has demonstrated face validity and excellent test-retest reliability in young children [47].

*Physical activity during school hours*. Children will be asked to wear ActiGraph GT9X Link accelerometers on their non-dominant wrist during school hours for five school days. Accelerometers will be distributed at the start of each school day (i.e., 9:00am) and collected at the end of each school day (i.e., 3:00pm). Physical activity will be categorised into light, moderate and vigorous intensity using validated cut-points [48].

*Cardiorespiratory fitness*. The 20m Multistage Fitness Test consists of one-minute stages of incremental speed running. Participants are required to run between two lines 20m apart whilst keeping up with the pace set by an audio recording. The test concludes upon voluntary exhaustion, or when they fail to reach the line concurrent with the audio signal on two successive occasions (warning provided by assessor). The participants are provided verbal encouragement during this test. Performance on this test has moderate-to-high criterion-related validity for estimating maximum oxygen uptake in children (r = 0.78, CI = 0.72 to 0.85) [49].

*Muscular power*. The Standing Long Jump Test requires the participant to stand behind a line and perform a maximal long jump–taking off and landing with two feet simultaneously. Participants perform two jumps, with the furthest jump recorded as the participants’ final score. This measure is associated with other lower body muscular fitness tests (R^2^ = .83 to .86) and with upper body muscular fitness tests (R^2^ = .69 to .85) in children [50], thus demonstrating its validity as a measure of overall muscular fitness in this population.

*Executive function*. Inhibition and task shifting will be assessed using two tests from the NIH Toolbox for the Assessment of Neurological and Behavioural Function (NIH-TB). Both tests are self-administered (not requiring administration by the researcher) and will be administered on an electronic tablet. For both tests, the versions designed for 8–11-year-old children will be used. The tests will be administered in groups, whereby up to 20 students will complete the tests on their tablets at the same time.

Inhibition will be assessed using the ‘Flanker Inhibitory Control and Attention Test’. This test is a measure of the ability to inhibit visual attention to irrelevant task dimensions. In children and adolescents, it has demonstrated convergent validity with ‘Delis-Kaplan Executive Function System–Inhibition (D-KEFS)’ and discriminant validity from the ‘Peabody Picture Vocabulary Test–Fourth Edition (PPVT-4)’ [51]. Test-retest reliability is high (ICC = .95, 95% CI: .92 to .97) in children and adolescents when tested between 7 and 21 days after initial assessment [51]. For each trial, the participant must pay attention to a central directional arrow. The arrow is flanked by other arrows which either point in the same (congruent) or different (incongruent) directions to the central arrow. The purpose of the task is to indicate which direction the middle arrow is pointing. The scoring algorithm integrates accuracy, a suitable measure in early childhood, and reaction time, a more relevant measure of adult performance on this task, and provides a score from 0 to 10. There are 40 trials and the average time to complete the task is 4 minutes.

To assess shifting (also referred to as ‘attention switching’, or ‘task-switching’), the ‘Dimensional Change Card Sort Test’ will be administered. This is a test of one’s ability to shift between multiple tasks, operations, or mental sets [52]. This test has demonstrated convergent validity with D-KEFS and discriminant validity from PPVT-4 in children and adolescents [51]. The test-retest reliability is high (ICC = .94, 95% CI: .92 to .96) [51]. For this test, participants are presented with a stimulus and instructed to match it to one of two stimuli either according to shape or to colour. Participants are presented with a ‘mixed’ block, whereby colour is relevant on the majority of trials with occasional, unpredictable shifts to shape. The words ‘colour’ or ‘shape’ appear on the screen and are presented audibly by the program before the presentation of the stimuli. The scoring is weighted towards accuracy for children and towards reaction time for adults. The test produces a score from 0 to 10 and takes approximately four minutes to complete the 40 trials.

#### Secondary outcomes (school champions)

Completed at baseline and follow-up.

*Teacher stress*. This will be assessed using the Teacher Stress Inventory [53]. Teachers will complete the 20-item self-report scale that uses a five-point Likert-type response format to measure occupational stress. This measure has demonstrated factorial validity as a measure of teachers’ stress [53].

*Teacher well-being*. This will be assessed using the Teacher Well-being Scale [54] This has demonstrated factorial validity as a measure of teacher well-being related to their workload, organisational well-being, and interactions with students [54].

#### Process evaluation

A detailed process evaluation will be conducted to determine schools’ implementation of the program:

*Acceptability and appropriateness*. At follow-up, *school champions* and *peer leaders* will be asked to report, via online survey and a face-to-face interview, the acceptability and appropriateness of the program and the support received to implement the program. Any adverse events or unintended consequences will also be collected at this time.

*Fidelity*. Project and school records, as well as post-intervention questionnaires completed by school principals, *school champions*, and *peer leaders* will be used to determine the schools’ implementation. We will use the following: (i) weekly schedule from the teachers to show that the 12 sessions were delivered (or how they were made up if they had to adapt); and (ii) during the observations, external change agents will collect data on *peer leaders’* adherence to the proposed session delivery.

*Implementation context*. To identify factors associated with implementation, at follow-up, school principals, and *school champions* will respond to items aligned with constructs from the Consolidated Framework for Implementation Research [55]; (i) *Inner setting* (e.g., compatibility with school values and direction), (ii) *Characteristics of the innovation* (e.g., perceived complexity and cost), and (iii) *Characteristics of the individual* (e.g., teachers’ knowledge, beliefs, and self-efficacy).

*School characteristics*. Data regarding the operational characteristics of schools, school participation in other physical activity programs, and implementation activity will be collected during a survey of school principals and *school champions*.

### Statistical analysis

General linear mixed models using the ‘lme4’ package in R software [56] will be used to assess the impact of treatment (L2L or control), time (treated as categorical with levels baseline, intervention end and follow-up) and the group-by-time interaction. The primary endpoint of the study will be intervention end (Term 3: weeks 5–10). Our analyses will be adjusted for the clustering of effects at the class and school levels, using random intercepts. Our mediation analyses will use a full information maximum likelihood procedure in Mplus. To determine whether changes in *peer leaders’* on-task behaviour mediate the effect of the L2L program on teachers’ work-related stress and wellbeing, structural equation models will be used to test the following: (i) the total effect of the program on work-related stress and well-being (C pathways); (ii) the effect of the intervention on the mediators (A pathways); (iii) the mediator effects on teachers’ work-related stress and well-being (B pathways); (iv) the direct effect of the L2L on teachers’ work-related stress and well-being with the inclusion of mediators in the model (Cʹ pathways) and (v) the indirect effect of L2L on teachers’ work-related stress and well-being (AB pathways).

To determine whether changes in *peers’* fundamental movement skills mediate the effect of the L2L program on their executive functioning, structural equation models will be used to test: (i) the total effect of the program on executive functioning (C pathways), (ii) the effect of the program on the mediators (A pathways), (iii) the mediator effects on executive functioning (B pathways), (iv) the direct effect of the program on executive functioning with the inclusion of mediators (C’ pathways), and (v) the indirect effect of the program on executive functioning (AB pathways). As Mplus does not support bootstrapping with clustered data, single-level bootstrap confidence intervals will be compared with confidence intervals adjusted for clustering.

Data regarding schools’ exposure to potential sources of contamination (or co-intervention) will be assessed via items in school, teacher, and Principal surveys following the intervention period. Potential effects on outcomes will be explored via sensitivity analyses.

### Data management plan

Once collected, the data will be saved locally on a university-issued laptop and backed up to a network provided by the university. The data will be regularly backed up to this drive via the setup of an automatic synchronization. The results of the study will not allow the identification of individual students, teachers or schools. Once the data have been collected, they will be deidentified using participant codes and entered into an electronic data file, questionnaires and other data collection sheets will be destroyed. Data will be stored for a minimum of five years on password protected files (only accessible to researchers).

### Ethics and dissemination plan

It is not expected that study participants will be at any greater risk of harm than they would be if they were to participate in other school-based physical activity. However, there is a section in the teacher handbook where adverse events may be reported. These handbooks are provided as part of the curricular materials described in Table 1. Any amendments to the study protocol will be made available via the Australian and New Zealand Clinical Trials Registry (trial number: ACTRN12621000376842). The findings of this RCT will be published in peer-reviewed journals. Regardless of the results, participating schools will receive a report outlining its findings at the conclusion of the trial. The data that support the findings of this study will be made available on request from the corresponding author, DRL.

## Discussion

There is consistent evidence that school-based, peer-led programs are beneficial for those students being led [6, 26, 57], though very few studies focus on the benefits for students leading these programs. Further, despite these programs being led by students, changes in students’ leadership ability are rarely examined as a study outcome. L2L is a multicomponent primary school-based leadership program that will focus on improving the leadership abilities of older children via their delivery of an FMS program to younger children. The development of L2L was informed by transformational leadership theory and our pilot study findings [24]. The findings from this program will generate new knowledge on the effectiveness of school-based leadership programs on students’ leadership, FMS, classroom behaviour, well-being, muscular fitness, physical activity, executive functioning, and the relationship between these outcomes.

## Supporting information

S1 Checklist SPIRIT checklist. (DOC)

S1 Fig Example of an L2L lesson plan. (TIF)

S1 File Full study protocol. (DOCX)
