# Supplementary figures and images for: Investigating the direct and indirect effects of a school-based leadership program for primary school students: Rationale and study protocol for the ‘Learning to Lead’ cluster randomised controlled trial
Source: PLoS One. 2023 Jan 20;18(1):e0279661. doi: 10.1371/journal.pone.0279661 (PMC9858303; doi:10.1371/journal.pone.0279661)

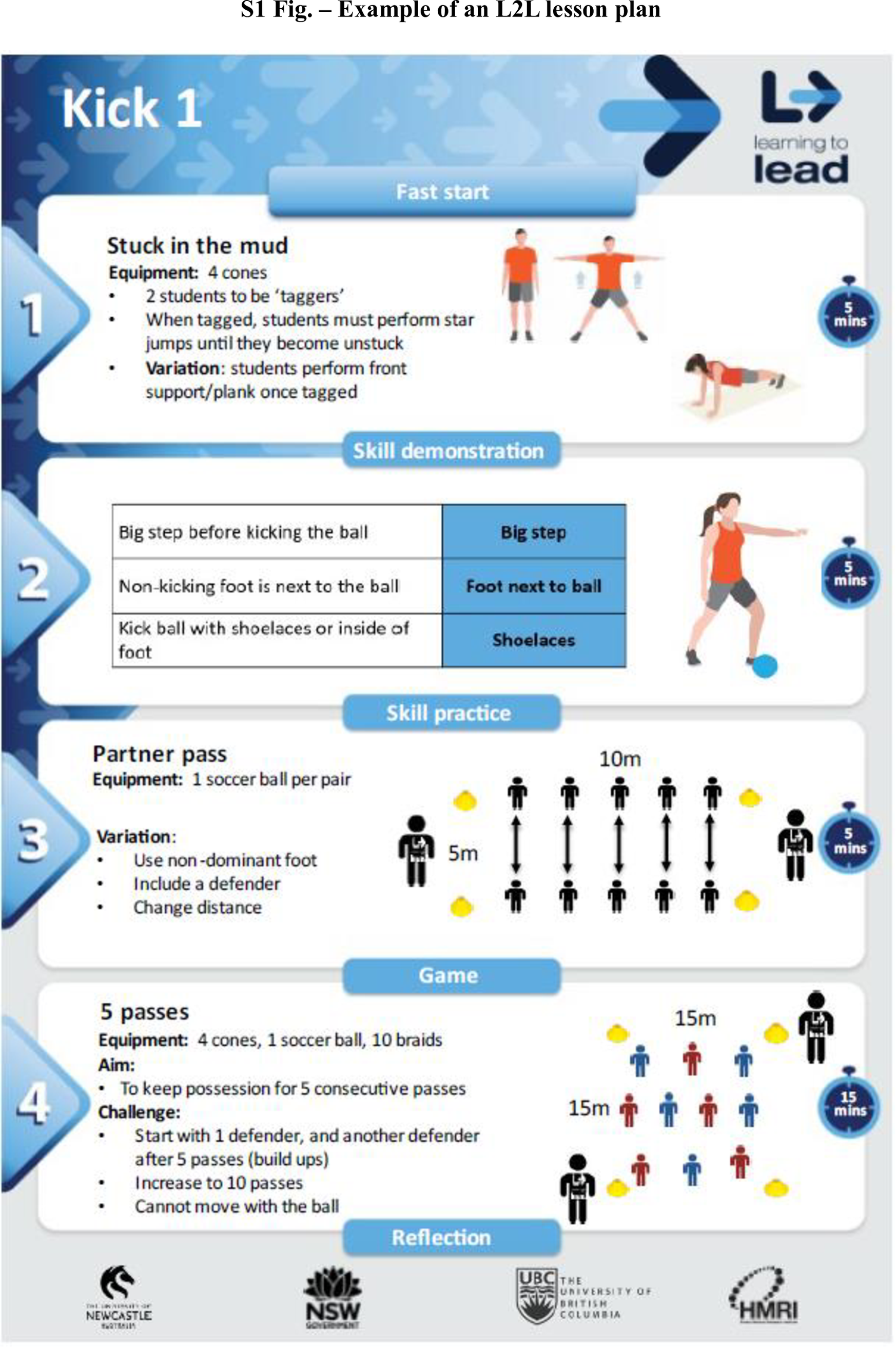

Supplement: S1 Fig — (TIF) [file pone.0279661.s002.tif]
