# Supplementary material for: Investigating the direct and indirect effects of a school-based leadership program for primary school students: Rationale and study protocol for the ‘Learning to Lead’ cluster randomised controlled trial
Source: PLoS One. 2023 Jan 20;18(1):e0279661. doi: 10.1371/journal.pone.0279661 (PMC9858303; doi:10.1371/journal.pone.0279661)
Supplement: S1 File — (DOCX) [file pone.0279661.s003.docx]

**PROJECT TITLE**

Investigating the direct and indirect effects of a school-based leadership program for students

**PROJECT AIMS AND BACKGROUND**

| ***The overall aim of our innovative project is to provide a comprehensive and original contribution to understanding the direct and indirect effects of a ‘multi-component’ leadership program for primary school students.*** Schools are ideal settings for developing students’ leadership skills, but there are few examples of evidence-based programs guided by leadership theory. This study will examine the effects of the Learning to Lead (L2L), a multi-component school program guided by transformational leadership theory on Grade 6 students’ leadership skills, self-efficacy and time ‘on-task’ in the classroom. Importantly, L2L has been designed in partnership with the NSW Department of Education (DoE), who have requested the program align with existing school-based leadership opportunities. Our research team will work with schools to ensure the tenets of transformational leadership theory are operationalised throughout the school. The program will provide an opportunity for Grade 6 students to develop their leadership skills by delivering physical activity sessions for Grade 3 students. As such, the proposed project will have the additional benefit of providing children with opportunities to be physically active at school and develop their movement skills, a valuable by-product given current rates of inactivity among youth. Australian teachers experience high levels of work-related stress and are looking for innovative strategies to manage student behaviour. Providing students with opportunities to develop their leadership skills may have ‘spill over’ effects on teachers’ work-related stress and well-being. The L2L program has been co-designed with the NSW DoE, thus maximising opportunities for dissemination. Considering there are over 1,600 government primary schools in NSW, attended by ~500,000 students, our proposed project has considerable potential for impact. Finally, enhancing children’s leadership skills may have long-term benefits for students’ educational and vocational trajectories. |
| --- |

**Schools are ideal settings for promoting life skills and health behaviours:** All primary school-aged children in Australia are required to attend school, which have the necessary equipment, personnel, facilities and curriculum to teach life skills and promote health behaviours. Leadership is an important life skill that can be developed in the school setting, and refers to the behavioural processes through which an individual influences others toward achieving specific goals or objectives^1^. Providing children with opportunities to develop their leadership skills may have benefits for the learning climate of the classroom (e.g., students’ behaviour during lessons) and other aspects of school life (e.g., behaviour during break time and extracurricular activities). Children’s leadership skills may also transfer to non-school settings and other life stages (e.g., employment in early adulthood). Surprisingly, the majority of school-based peer leadership programs have focused on peers’ (i.e., those being led) health behaviours and outcomes^2,3^, rather than identifying potential benefits for the peer leaders themselves. Moreover, few school-based leadership programs have been guided by leadership theory^2,3^.

**Transformational leadership theory:** Although there are many published leadership frameworks, transformational leadership theory (not to be confused with transformative leadership)^4^ has the strongest evidence base^5,6^. Research conducted in a variety of settings has demonstrated that transformational leadership is associated with a range of positive outcomes, including higher levels of empowerment^7^, motivation^8^, and performance^9^. Transformational leaders demonstrate behaviours that empower and inspire others, they transcend their own self-interests, and provide others with the confidence to achieve high levels of functioning^4^. Transformational leadership theory consists of four inter-related behavioural dimensions: (i) *Idealised influence*- fostering trust and respect by role modelling ideal behaviour, (ii) *Inspirational motivation*- displaying optimism, enthusiasm, and having high expectations for others, (iii) *Intellectual stimulation-* encouraging others to consider issues from a different perspective, and (iv) *Individualised consideration*- recognising and supporting others’ physical and psychological needs. Of note, **PI Beauchamp** is leading a program of research focused on extending transformational leadership theory to schools and more specifically, to physical education (PE). For example, he has previously demonstrated that PE teachers can become more ‘transformational’, which in turn predicts students’ enjoyment of PE and their activity beyond the classroom^10^. Of note, the appropriateness of transformational leadership theory for education settings was highlighted in a seminal review by Slavich and Zimbardo^11^, who described **PI Beauchamp’s** work as “an elegant series of studies”.

**Physical inactivity is a national problem:** Participation in moderate-to-vigorous physical activity (MVPA) is essential for children’s physical, social, psychological and cognitive development^12,13^. Despite the extensive benefits, less than 20% of Australian children are sufficiently active^14^. Of additional concern, many children leave primary school without mastering basic movement skills (e.g., throwing, catching, running and kicking)^14^. While schools represent an ideal context to enhance children’s activity levels and promote movement skill competencies, intervention effects are typically modest. For example, **CI Lubans** and colleagues have reported that the average effect of school-based physical activity interventions on objectively measured physical activity is less than 2 minutes of MVPA/day^15^. Poor implementation by teachers is a major factor explaining the minimal effect of school-based interventions^16^. This interpretation is further strengthened when examining the ‘program drift’ and ‘voltage drop’^17^ that occur when interventions progress from efficacy testing (which typically involve high levels of support from research teams) to effectiveness and dissemination (when support is often removed)^18,19^. Moreover, many school-based physical activity interventions are too complex to implement by ‘time poor’ teachers. As noted by **CI Nathan** and colleagues^20^, lack of time, support, and perceived teacher competence appear to be the major barriers to the implementation of physical activity interventions in schools.

**Aligning student leadership opportunities with physical activity promotion may help teachers:** Australian teachers report high levels of work-related stress and burnout^21^. Sources of stress for school teachers include managing student discipline, high workloads, poor working conditions, and lack of support from management^22,23^. A recent study of Australian teachers (N = 960) found new measures for managing student behaviour were among teachers’ top priorities for change to improve their own well-being^24^. Providing students with opportunities to develop leadership skills may help to manage students’ behaviour within and beyond the classroom. Transformational leadership provides a useful framework for guiding student behaviour that may have ‘spill over’ effects on teachers’ well-being. To our knowledge, no previous study has tested the effects of a school-based leadership program on students’ on-task behaviour in the classroom. Previous investigations have demonstrated that peer leadership programs can have a range of benefits for those being led, such as improvements in physical activity levels^25^, nutrition education^26^, and drug use^27^.

**Pilot work completed by our research team:** We conducted a pilot study to evaluate a school-based peer leadership program in two primary schools in NSW (N = 224 students)^28^. Delivered by **CI Lubans,** the intervention resulted in large improvements in *Peer leaders’* effectiveness (*d* = 1.09) and *Peers’* movement skill competency (*d* = 0.95). Importantly, the program was well received by teachers and students, increasing the likelihood of program dissemination. However, our pilot study involved a quasi-experimental design in two schools and our findings need to be confirmed using a robust cluster randomised controlled trial (RCT) design.

**INVESTIGATOR(S)/ CAPABILITY**

**Research opportunity and performance evidence**. Our multidisciplinary research team includes outstanding researchers in health education (**CIs Lubans, Smith**), psychology (**PI Beauchamp**) and public health (**CI Nathan**). We have a proven track record and international standing in school-based research, having conducted more than 50 school-based RCTs. **CI Lubans** is an NHMRC Senior Research Fellow, former ARC Future Fellow and was a member of the 2018 Guideline Development Group for the Australian 24-hour Movement Guidelines for Children and Young People. **PI Beauchamp** is an international expert in the psychology of health behaviour change and has led several RCTs, including those that focus on leadership development and health outcomes. **CI Nathan** is a full-time Medical Research Future Fund Research Fellow, with an emerging international reputation in implementation science. **CI Smith** is a mid-career researcher with expertise in Physical Education and the design of school-based physical activity interventions.

**Time and capacity to undertake the research:** The research team has the necessary time and expertise to conduct this innovative study. **CI Lubans** is an NHMRC Senior Research Fellow and former ARC Future Fellow within the University of Newcastle’s (UoN) Priority Research Centre for Physical Activity and Nutrition (PRC-PAN). He will lead the overall coordination and evaluation of the study. **PI Beauchamp** is a Professor of Exercise and Health Psychology in the School of Kinesiology at the University of British Columbia (UBC). He will guide intervention development in relation to transformational leadership. **CI Nathan** is a Conjoint Senior Lecturer in the School of Medicine and Public Health at UoN and will guide the L2L implementation framework and evaluation. **CI Smith** is a Senior Lecturer in the School of Education and co-deputy lead of the PRC-PAN school-based research theme. He will lead the development of curricular materials.

**Experience in training, mentoring and supervision: CI Lubans** and **PI Beauchamp** have extensive experience in research training and mentoring. They have demonstrated a commitment to supporting their students’ careers by providing them with: (i) networking opportunities through collaborations with international colleagues, (ii) high quality publications, including first authorship on major publications (e.g., *SCORES-* Cohen et al 2015, *Med Sci Sports Exerc*; *NEAT-* Dewar et al 2013 *Am J Prev Med*) and (iii) nominations for national research awards**. CI Lubans** has supervised 16 PhDs, 2 MSc and 6 Honours students to completion. In 2017, he received the UoN Faculty of Education and Arts Vice Chancellor’s Award for Research Supervision Excellence (based on testimony from 10 PhD students). **PI Beauchamp** has supervised 15 graduate students to completion. **CI Nathan** currently supervises 5 PhD students and has 2 completions. **CI Smith** has 1 PhD and 2 Honours completions.

**Capacity of team to build collaborations within Australia and beyond.** Our research team has extensive collaborations with researchers in Australia and internationally. In addition, we have established strong collaborative relationships with partner organisations, such as the NSW DoE and NSW Health. These partnerships have supported the dissemination of numerous school-based physical activity programs in NSW schools. For example, **CI Lubans** partnered with Australian Catholic University and the NSW DoE to secure an NHMRC partnership grant (APP1114281 for $1.3 million) to evaluate and disseminate a multi-component school-based physical activity program, known as *iPLAY* in more 150 primary schools in NSW. **CI Nathan** has received two NHMRC partnership grants with the NSW DoE (~$2 million) to implement school physical activity programs.

**PROJECT QUALITY AND INNOVATION**

## **Contribution to an important gap in knowledge**

Our project is guided by transformational leadership theory^4^ and existing evidence that transformational leaders can motivate others to do more than what they thought was originally possible^5^. Research conducted in different settings (e.g., workplaces) has demonstrated that transformational leadership is linked with a range of positive outcomes for those being led.^7-9^ Our research team has previously demonstrated that: (i) teachers can learn to utilise transformational teaching practices and inspire adolescents to be more physically active^10^; and (ii) Grade 6 students can become transformational leaders in their schools and improve younger children’s fundamental movement skill (FMS) competency^28^. We have also received funding to adapt the L2L program for Canadian schools and test the effects on children’s physical literacy (i.e., motivation, perceived competence, self-concept, and FMS competence) in the *Peer Leadership for Physical Literacy* trial. The proposed project will build upon these findings and be the first to test the hypothesis that improving children’s leadership skills may have ‘spill over’ effects for leaders’ time ‘on-task’ in the classroom and teachers’ work-related stress and well-being (Figure 1). More specifically, we hypothesise that improvements in leaders’ time ‘on-task’ in the classroom will mediate the effect of the program on teachers’ work-related stress and well-being. There is evidence to suggest that negative student-teacher relationships are associated with lower occupational well-being^29^. Moreover, new measures for managing student behaviour have been identified by teachers as a top priority to improve their own well-being^24^. ***Of note, teachers involved in our pilot study reported improvements in students’ behaviour following participation in our leadership program****.* Finally and based on our previous work^28^, we also hypothesise that children (i.e., those being led), will experience improvements in school-based physical activity, actual and perceived FMS competency.

**Figure 1: Conceptual model illustrating the potential direct and indirect effects of the L2L program**


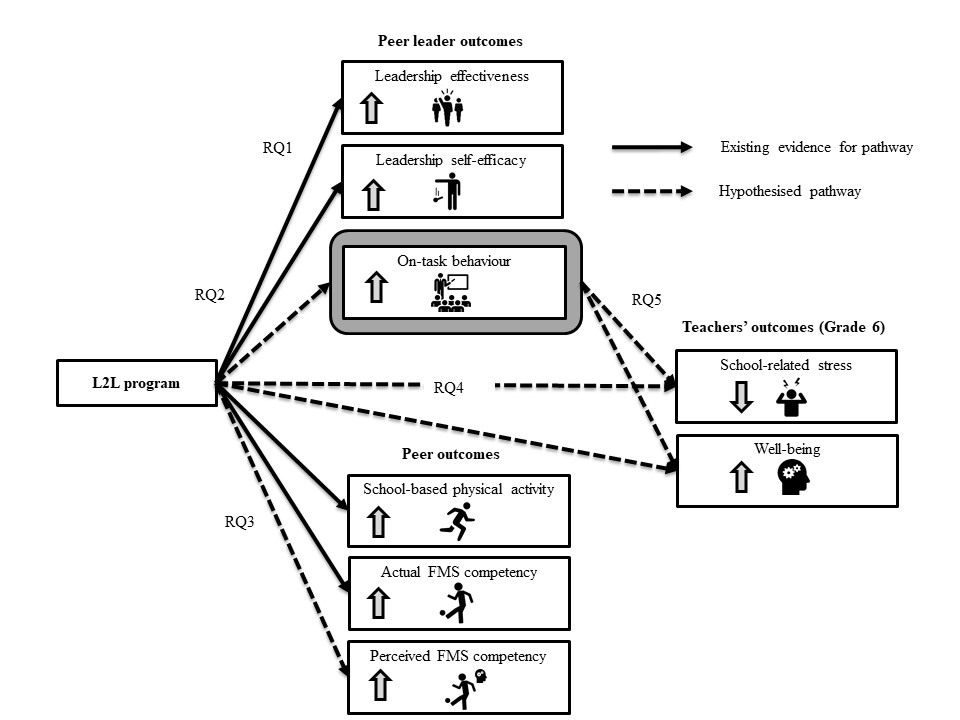


**Aim and research questions**

The overall aim of our innovative project is to provide a comprehensive and original contribution to understanding the direct and indirect effects of a school-based leadership program for primary school students. In partnership with the NSW DoE School Sport Unit, we will evaluate the ‘Learning to Lead’ (L2L) program, which aims to: (i) enhance Grade 6 students’ (*Peer leaders*) leadership skills, leadership self-efficacy, and time on-task in the classroom; (ii) increase Grade 3 students’ *(Peers)* physical activity levels, actual and perceived fundamental movement skill competency; and (iii) decrease teachers’ work-related stress and improve their well-being. More specifically, we will answer the following research questions (RQs):

RQ1) What is the impact of the L2L program on the primary outcome, *Peer leaders’* leadership effectiveness?

RQ2) What is the impact of the L2L program on the secondary outcomes, *Peer leaders’* leadership self-efficacy and time ‘on-task’ in the classroom?

RQ3) What is the impact of the L2L program on secondary outcomes, including *Peers’* school-based physical activity, actual and perceived FMS competency?

RQ4) What is the impact of the L2L program on teachers’ work-related stress and well-being?

RQ5) Do changes in *Peer leaders’* on-task behaviour mediate the effect of the L2L program on teachers’ work-related stress and well-being?

## **Research design**

We will evaluate the *‘Learning to Lead’* program using a two-arm parallel group cluster randomised controlled trial (RCT) with an intervention group and a control group. Assessments will be conducted at baseline (Term 1, weeks 5-10), mid-intervention (Term 3, weeks 5-10) and follow-up (Term 4, weeks 5-10), and the study will be conducted in two cohorts (2022 and 2023). The trial will be registered with the Australian New Zealand Clinical Trials Registry and the design, conduct and reporting will adhere to CONSORT (Consolidated Standards of Reporting Trials)^30^ and TIDieR (Template for Intervention Description and Replication)^31^ checklists. Starting in Term 4, 2021 (Table 1), we will gain university ethics and NSW DoE’s State Education Research Applications Process (SERAP) approval. We will refine program materials in 2021 and the trial will commence in 2022.

## **Setting and participants**

*i) Schools and teachers:* Government primary schools located in Metropolitan South Sydney will be eligible to participate (N = 359 schools). Schools will be eligible to participate if they have at least two Grade 6 classes and are not participating in any other leadership or physical activity programs and have provided a written expression of interest. In 2022 (Cohort 1), we will randomly select 10 schools from those that have expressed interest to participate in the study and identify up to 10 replacement schools. Once we have 10 schools, we will match pair schools according to their size and level of socio-economic disadvantage (using My School data)^32^. We will follow the same process in 2023 (Cohort 2), until we reach our total sample size of 20 schools. Principals will provide written informed consent. We will recruit teachers of Grade 6 and Grade 3 students. We will use evidence-based recruitment and retention strategies to maximise participation and minimise drop-out (at both school and teacher levels). This will include pre-notification, the use of a dedicated recruitment co-ordinator (i.e., Project Manager), and repeated reminders and regular contact with schools. Previous studies conducted by our research team have achieved teacher participation and school attrition rates of >80% and <20%, respectively^33,34^. The provision of teaching materials and the alignment of professional learning with the *NSW Education Standards Authority* accreditation process will incentivise teachers to participate.

*ii) Students:* Two Grade 6 and two Grade 3 classes from each school will be invited to participate. If schools have more than two Grade 6 or Grade 3 classes, classes will be randomly selected. Children from other grades (i.e., Grades 1, 2 and 4) may participate in the program, but they will not be involved in the data collection. We will recruit ~800 Grade 6 students and ~800 Grade 3 students. Parents of students from Grade 6 and 3 will be required to provide written informed consent and students will provide assent. Henceforth, Grade 6 students will be referred to as *Peer leaders,* while Grade 3 students will be referred to as *Peers.* We have achieved high rates of student consent in our previous school-based physical activity studies (typically ~80%^28,33,34^).

## **‘Learning to Lead’ (L2L) program**

**Development and theoretical framework:** L2L is a ‘multi-component’ school program co-developed with an advisory group consisting of key stakeholders, education experts, physical activity experts, teachers, and school principals. The program is informed by the tenets of transformational leadership theory^5^ and based on previous interventions designed by our research team^28,34^. Importantly, key stakeholders have recommended the L2L program align with existing school-based leadership opportunities. As such, our research team will work with schools to ensure the tenets of transformational leadership theory are operationalised throughout the school. For example, students elected onto the Student Representative Council will participate in a brief induction program guided by transformational leadership theory. Schools will also be provided L2L posters explaining and promoting the transformational leadership principles. The L2L program is guided by the conceptual framework developed by Kelloway and Barling^35^ and refined by **PI Beauchamp**^5^. This framework involves the following components: (i) explanation of transformational leadership behavioural principles, (2) demonstration of leadership behaviours using real-world examples that are relevant for children, (3) providing opportunities for children to practice the leadership behaviours, (4) receiving feedback on the implementation of leadership behaviours, and (5) development of self-regulatory strategies to support sustained implementation. Integral to this framework is the opportunity for *Peer leaders* to intellectually engage with the meaning of ‘leadership’ and how their own behaviours can influence others in positive and (if not enacted appropriately) negative ways.

**Leaders’ involvement in the program:** Grade 6 teachers will deliver 7 x 40-minute lessons on the program to *Peer leaders* in Term 2. The seven lessons will include a mixture of practical activities and information to support leaders to deliver the program. It will address the four dimensions of transformational leadership ^4^. These include: (i) *Idealised influence,* (ii) *Inspirational motivation,* (iii) *Individualised consideration,* and (iv) *Intellectual stimulation.* To present this framework in age-appropriate terms, we refer to the four leadership dimensions as*: i) Role modelling, ii) Motivating others, iii) Considering others,* and *iv) Helping others to think.* Leaders will also be taught the correct movement skill patterns for five object control skills (i.e., catch, underarm throw, overarm throw, bounce, and kick). *Peer leaders* will be provided with models of the ideal structure of a fundamental movement skill session which includes: (i) *Brief skill introduction*, (ii) *Warm-up game*, (iii) *Skill development* with key teaching points, (iv) *Skill application* in a small-sided game and (v) *Closure*. *Peer leaders* will be trained to provide basic teaching cues, individualised feedback, and opportunities to practice movement skills in a supportive, fun, and encouraging environment. Intervention schools will be provided with a class set of age-appropriate and purpose-designed laminated “lesson-plans” for leaders to use. While *Peer leaders* will be provided with resources and a recommended session structure, they will have guided autonomy in their delivery of sessions. Based on feedback from our initial pilot study^28^, leaders will be provided with some simple behaviour management techniques to assist managing children’s behaviour. ***We are unaware of any school leadership programs that provide children such an extensive opportunity to develop their leadership skills.***

**Peers’ involvement in the program:** *Peers* (i.e., students in Grade 3) will be placed into groups of 10–12 students by their classroom teachers. Two *Peer leaders* will be allocated to each group, who will deliver 2 x 30-min fundamental movement skill sessions per week for 10-weeks (1 school term). In line with transformational leadership principles, *Peer leaders* will remain with the same *Peers* for the 10-week duration.

**Teachers’ involvement in the program:** Grade 6 teachers will be provided with training and support to facilitate *Peer leaders’* delivery of the FMS sessions. Teachers will be provided with all curriculum materials to deliver the program in their schools. At the end of each session, Grade 6 teachers will be encouraged to meet briefly with *Peer leaders* to discuss any challenges, concerns and offer potential solutions. This will also provide an opportunity for teachers to provide general and specific feedback to *Peer leaders.* *Of note, teachers in our pilot study indicated that the program reduced their burden because (i) detailed lesson plans were provided and (ii) older students assumed some responsibility for curriculum delivery.*

**Implementation support:** To support schools’ implementation of the L2L program, we will use a range of evidence-based implementation strategies:

1. *School champions:* Two school champions (Grade 6 teachers), will be identified and recruited to act as organisers at each of the study schools. These teachers will liaise with the research team to recruit students (leaders and children), organise study assessments and teacher workshops. Following their training school champions will deliver a 20-minute overview of the program to their colleagues. This presentation (delivered by school champions) will provide a rationale for the L2L program and provide other teachers in their school with ways they can support the program and the *Peer leaders*.
2. *External change agents:* Each school will be allocated an external change agent to support program implementation. The change agents, from the NSW DoE School Sport Unit or the University of Newcastle (UoN), will provide training and on-going support to school champions. In addition, the change agent will observe two sessions per school over the 10-week study using a structured observation checklist. This information will be provided back to teachers, who will then share this information privately with leaders.
3. *Professional learning workshop:* To enable school champions to deliver the 7 x 40-minute lessons professional learning for school champions will be provided as a full-day workshop registered with the NSW Educational Standards Authority. At the end of the workshop teachers will complete an action plan addressing potential barriers and facilitators to program implementation.
4. *Curricular materials:* School champions will be provided with all the curricular materials to deliver the program at the professional learning workshop. The following materials will be provided to schools: L2L unit of work and lesson plans, lesson materials, film clips, experiential activities and games. All curricular materials will be aligned with the Stage 3 Personal Development, Health and Physical Education curriculum. More specifically, the content will align with the: (i) Movement Skill and Performance, and (ii) Healthy, Safe and Active Lifestyles strands.
5. *Equipment and resources:* Schools will be provided with a sports equipment pack (i.e., balls, marker cones, paddle bats etc.) to assist in the delivery of the program (~$1,000 AUD). Schools will also receive posters explaining the transformational leadership principles to be displayed in classrooms.

**Control group**

To prevent compensatory rivalry and resentful demoralisation, we will use a wait-list control group. Schools allocated to the control group in cohorts 1 and 2 will receive the program in 2022 and 2023, respectively. Data regarding schools’ exposure to potential sources of contamination (or co-intervention) will be assessed via items in school, teacher, and Principal surveys. Potential effects on outcomes will be explored via sensitivity analyses.

## **Primary and secondary outcomes**

**Measures and data collection:** All assessments will be conducted in the study schools by trained research assistants blinded to condition allocation. Questionnaires will be completed in exam-like conditions using an online survey with electronic tablets. Standard demographic information will be collected at baseline.

**Primary outcome (*Peer leaders*):** Completed by teachers at baseline, mid-intervention and follow-up:

1. *Leadership effectiveness:* Grade 6 teachers will be asked to rate students’ leadership skills using an adapted version of the Transformational Teaching Questionnaire^36^ that assesses students’ displays of individualised consideration, inspirational motivation, intellectual stimulation, and idealised influence. The four-item measure utilises the common stem: ‘The student that I’m rating’... The four-item composite measure of leadership effectiveness was found to have acceptable internal consistency in our pilot (α = 0.89 to 0.92).

**Secondary outcomes (*Peer leaders*):** Completed at baseline, mid-intervention and follow-up:

1. *Leadership self-efficacy*: *Peer leaders* will be asked to complete an 11-item measure based on standard protocol for assessing self-efficacy beliefs^37^. *Peer leaders* will be asked to rate their confidence to perform the key leadership behaviours being targeted in the intervention. Responses to items are provided on a 0-100 scale (at 10-point increments) anchored by 0% (“No confidence”), 50% (“Somewhat confident”), and 100% (“Completely confident”). The items were adapted from a previous questionnaire^38^ with example items including “…be a role model to other students”, “…teach physical activity skills to other students”, “…help students feel safe about joining in”.
2. *Time-on*-*task in the classroom:* Classroom observations will be conducted by trained research assistants at baseline (Term 1: weeks 5-10), mid-intervention (Term 3: weeks 5–10) and follow-up (Term 4: weeks 5-10) using established methods^39^. To increase standardisation across schools, the observations where possible, will take place during mathematics lessons at the same time of day. During each 30 min observation period (starting 5 min after students enter the classroom), research assistants will assess the on-task and off-task behaviour of six randomly selected students (5 min per student). For each lesson, two observers will randomly select 6 boys and 6 girls (i.e., 12 students in total) and the order in which they are observed (teachers and students will not know who is being observed). Observers will listen to an audio file via headphones, which will inform them when to observe and record (in 15 second intervals). After each 15 second interval, the observers will record the student's behaviour by circling an appropriate code (i.e., actively engaged, passively engaged, off-task motor, off-task verbal or off-task passive) using an observation sheet. Time spent on- and off-task during the lesson will be expressed as a percentage of total lesson time. **CI Lubans** has extensive experience in conducting classroom observations^40,41^.

**Secondary outcomes (*Peers*):** Actual and perceived object control fundamental movement skill (FMS) competency will be assessed at baseline and follow-up. Physical activity will be assessed at baseline and mid-intervention. Our research team has substantial expertise in the assessment of FMS and physical activity^33,34,42^.

1. *Actual FMS competency*: Will be assessed using a subset of object control skills (i.e., underarm throw, overarm throw, kick, catch, and bounce) from the Test of Gross Motor Development 3^43^. Children will be filmed performing two trials of each of the 5 skills. These skills were selected due to their transferability into a variety of different sports that are popular among Australian children. Research assistants, blinded to group allocation, will assess the videos according to the performance criteria. Each skill component will be scored a “1” if observable and performed correctly or “0” if performed incorrectly. This procedure will be completed for each of the two trials, and trial scores will be summed to calculate a total score for each skill. An overall object control movement skill score will be calculated by summing the skill scores.
2. *Perceived FMS competency:* Perceived competence in the same 5 fundamental movement skills described above will be assessed using the Pictorial Scale of Perceived Movement Skill Competence^44^, which has acceptable validity and reliability in young children.
3. *Physical activity during school hours:* Children will be asked to wear ActiGraph GT9X Link accelerometers on their non-dominant wrist during the school day for one week. Accelerometers will be distributed at the start of each school day (i.e., 9:00am) and collected at the end of each school day (i.e., 3:00pm). Physical activity will be categorised into light, moderate and vigorous intensity using validated cut-points^45^. Children will wear accelerometers on two occasions: (i) Term 1 (weeks 5-10): baseline, (ii) Term 3 (weeks 5-10): mid-intervention, (iii) Term 4 (weeks 5-10): follow-up.

**Secondary outcomes (Teachers):** Completed at baseline and follow-up only.

1. *Teacher Stress Inventory*^46^: Teachers will complete the 20-item self-report scale that uses a five-point Likert-type response format to measure occupational stress.
2. *Teacher Well-being Scale^47^*: This measure is focused on teacher well-being related to workload, organisational well-being, and interactions with students. **CI Nathan** has assessed teachers’ stress and well-being in a recent study with ~300 teachers.

**Process evaluation:** A detailed process evaluation will be conducted to determine schools’ implementation of the program:

1. *Acceptability and appropriateness*: At follow-up, intervention Principals, school champions, teachers and leaders will be asked to report, via paper-based survey, the acceptability and appropriateness of the program and the support received to implement the program. Any adverse events or unintended consequences will also be collected at this time.
2. *Fidelity*: Project and school records, as well as post-intervention questionnaires completed by intervention Principals, school champions, teachers and leaders will be used to determine the proportion of schools that received and utilised the implementation strategies. We will use the following: (i) Weekly sign-on sheets completed by leaders; (ii) Weekly schedule from the teachers to show that the 2 sessions were delivered (or how they were made up if they had to adapt); (iii) Leaders attendance at the training delivered by the school champion/Grade 6 teacher, and (iv) During the observations, external change agents will collect data on leaders’ adherence to the proposed session delivery.
3. *Implementation context:* To identify factors associated with implementation, at follow-up, Principals, school champions and teachers will respond to items aligned with constructs from the Consolidated Framework for Implementation Research^48^; (i) *Inner setting* (e.g., compatibility with school values and direction), (ii) *Characteristics of the innovation* (e.g., perceived complexity and cost), and (iii) *Characteristics of the individual* (e.g., teachers’ knowledge, beliefs, and self-efficacy).
4. *School characteristics*: Data regarding the operational characteristics of schools, school participation in other physical activity programs, and implementation activity will be collected during a survey of school principals and classroom teachers.
5. *Intervention costs*: We will collect information regarding the cost of the intervention delivery, including training, equipment and support.

**Sample size:** To ensure 80% power to detect a conservative effect of *d* = 0.50 for leadership effectiveness (adjusted between-group difference of ∼0.4 units with standard deviation of 0.8), we required 128 *Peer leaders* for a non-clustered trial (two-tailed, *p*<0.05). However, as recommended by CONSORT^30^, we adjusted our power calculation for the clustering of effects at the school level using a correction factor of [1+ (m − 1) × ICC], where m = participants per school and ICC = intraclass correlation coefficient. Assuming an average class size of 20 participating students, two classes per school and an ICC for leadership of 0.15, the correction factor is 5.68 [i.e., 1 + (40−1) × 0.15]. The required sample size to achieve 80% power with α levels set at *p*<0.05 is 727 (i.e., 128 x 5.68). Assuming each school has approximately 60 x Grade 6 students (2 classes), with a conservative response rate of 67% and allowing for an expected 10% loss to follow-up, we will aim to recruit at least 40 Grade 6 students from each of 20 schools (N = 800). We will also recruit 40 Grade 3 students per school (N = 800).

**Statistical analysis:** Research questions 1 to 4 will be answered using linear mixed models in IBM SPSS Statistics for Windows, V.20.0 (IBM, Armonk, New York, USA). The models will be used to assess the impact of treatment (‘Learning to Lead’ or control), time (treated as categorical with levels baseline, mid-intervention and follow-up) and the group-by-time interaction. The primary end-point of the study will be follow-up (Term 4, weeks 5-10). Our analyses will be adjusted for the clustering of effects at the class and school levels, using random intercepts. Research question 5 will be answered using a full information statistical maximum likelihood procedure in Mplus, Version 8 that uses all available data. Structural equation models will be used to test the following: (i) the total effect of the program on work-related stress and well-being (C pathways); (ii) the effect of the intervention on the mediators (A pathways); (iii) the mediator effects on teachers’ work-related stress and well-being (B pathways); (iv) the direct effect of the L2L on teachers’ work-related stress and well-being with the inclusion of mediators in the model (Cʹ pathways) and (v) the indirect effect of L2L on teachers’ work-related stress and well-being (AB pathways). As Mplus does not support bootstrapping with clustered data, single-level bootstrap confidence intervals will be compared with confidence intervals adjusted for clustering.

**Potential to enhance international collaboration:** This project will further strengthen the existing alliance between the UoN and the UBC. **CI Lubans** and **PI Beauchamp** have an extensive history of collaboration, including multiple research visits to Vancouver and Newcastle and 11 co-authored peer reviewed journal articles. All investigators listed on this grant have worked together on externally funded research projects.

**BENEFIT**

**Generation of new knowledge:** Our project is uniquely positioned to advance knowledge in the following areas:

1. High quality evidence for the effectiveness of a school-based leadership program. Our pilot study demonstrated the feasibility and preliminary efficacy of a school-based leadership program. Our proposed study, using a cluster RCT design, will provide robust evidence for program effectiveness.
2. Impact of a transformational leadership program on students’ time-on-task in the classroom. Feedback provided by teachers involved in our pilot study^28^ suggests that participating in a leadership program guided by transformational leadership theory improves students’ behaviour in the classroom. However, this will be the first study to test this hypothesis in a rigorous cluster RCT. We hypothesise that teaching students about the importance of transformational leadership principles (i.e., role modelling, motivating others, considering others, and helping others to think), will have a ‘spill over’ effect onto their time ‘on-task’ in the classroom.
3. Impact of a transformational leadership program on teachers’ well-being and work-related stress. Poor student behaviour is a major source of stress for teachers. Negative student-teacher relationships are associated with high levels of stress and lower occupational well-being in teachers^29^. New strategies for managing student behaviour has been identified by teachers’ as a top priority for change to improve their well-being^24^. This will be the first study to test the effect of a transformational leadership program on teachers’ work-related stress and well-being. We hypothesise that improvements in students’ leadership effectiveness and time ‘on-task’ will mediate the effect of the program on teachers’ reduced work-related stress and improved well-being.

**Benefits for Australia and international communities:** The proposed project will have benefits for Australia across multiple domains. First, transformational leadership is associated with a range of positive outcomes for leaders and those being led, including higher levels of empowerment^7^, motivation^8^, and performance^9^. Providing children with opportunities to develop their leadership skills may have short-term benefits for students and schools. It may also have medium- and subsequent long-term benefits for students’ academic performance and study or employment opportunities. As noted in the Australian Institute for Teaching and School Leadership’s Strategic Plan 2019–2022, ‘Leadership is a team effort at all levels’. Second, Australian teachers report high levels of work-related stress^21^ and nearly one in three consider leaving in their first five years of employment^49^. Providing teachers with innovative methods to manage their students’ behaviour may help reduce the financial burden of teacher burnout. Finally, it has been estimated that the annual economic burden of physical inactivity in Australia is $555.6 million^50^. Less than 20% of Australian children are sufficiently active^14^ and schools are ideal venues to address this challenge. Physical activity promotion is aligned with a number of national and international recommendations and priorities, including: (1) The National Strategic Framework for Chronic Conditions, Objective 1: Focus on Prevention for a Healthier Australia (i.e., Strategic Priority Area 1.1 physical and social environment determinants), (2) Sport 2030 Strategic Priority ‘Building a More Active Australia’ which aims to ‘Reduce inactivity amongst Australians by 15% by 2030’, and (3) World Health Organization’s Global Action Plan on Physical Activity 2018–2030 (e.g., ‘Create Active People’). As such, this project will contribute to the Science and Research Priority Area of ‘Health’.

**FEASIBILITY**

**Suitability of the research environment and availability of necessary facilities.** This research project will be located in the UoN’s PRC-PAN. In the Excellence in Research for Australia assessment, our Priority Research Centre achieved a 5 (i.e., well above world standard) for Human Movement and Sports Science for three consecutive assessment rounds (2012, 2015 and 2018). In practical terms, this means our team has access to a knowledge base, education and academic networks, specialised equipment (e.g., iPads, accelerometers and digital video recordings for analysing students’ motor skill proficiency), and administrative support all co-located.

**Experience of the research team to complete the project.** Over the past 15 years, our team has conducted >50 school-based experimental studies, involving over 300,000 children and adolescents. We have achieved high consent rates from both schools and students using evidence-based strategies, such as: 1) promotion of research to relevant principals, teachers, parents, and students; 2) dissemination of study information using methods allowing direct contact with parents; 3) provision of incentives to teachers and students; 4) making reminder contact. Recent examples: 1,219/1741 students (70%) from 22 schools in *iPLAY*; 1,233/1,468 students (84%) from 10 schools in *Physical Activity 4 Everyone*^33^; and 174/176 students (99%) from 2 schools in *GLASS*^28^- the pilot for this proposal. Finally, it is important to note that the NSW DoE has been heavily involved in the development of this research project. Improving children’s leadership skills is clearly aligned with the NSW DoE’s School Excellence Framework^51^, which states- “*At the other end of schooling, teachers and schools support students to make successful transitions to future learning and employment, with the skills to make informed contributions as citizens and leaders*”. The NSW DoE have been working, and will continue to work, closely with the research team on all aspects of program development and will have an on-going role through participation in a Project Steering Committee. Study timeline and milestones provided below (Table 3).

**Table 3: Study timeline and milestones**

| **Milestones** | **2022** | | | | **2023** | | | | **2024** | | | |
| --- | --- | --- | --- | --- | --- | --- | --- | --- | --- | --- | --- | --- |
|  | **School terms** | | | | **School terms** | | | | **School terms** | | | |
|  | T1 | T2 | T3 | T4 | T1 | T2 | T3 | T4 | T1 | T2 | T3 | T4 |
| Intervention development and ethics approval |  |  |  |  |  |  |  |  |  |  |  |  |
| Cohort 1: school recruitment (T1), cluster randomised controlled trial (T1-4) |  |  |  |  |  |  |  |  |  |  |  |  |
| Cohort 2: school recruitment (T1), cluster randomised controlled trial (T1-4) |  |  |  |  |  |  |  |  |  |  |  |  |
| Analysis of data (T1), publication of findings (T2-3), program dissemination (T4) |  |  |  |  |  |  |  |  |  |  |  |  |

**COMMUNICATION OF RESULTS**

We will utilise a variety of strategies to communicate our results to other researchers, relevant stakeholders and the broader community. First, to maximise the reach of our research we will publish a small number of high quality peer-reviewed journal articles across multiple fields of research (e.g., *Educ Psych Rev*, *Teach Teacher Educ*, and *Bri J Sports Med).* Second, we will utilise the internal communication channels of the NSW DoE to disseminate findings via online newsletters, department-wide email, and social media channels. Third, the general public will be informed through media releases via the UoN, while reports will be made available through the UoN’s online digital repository. Fourth, our research findings will be communicated to the academic community through a series of either virtual or in-person (pending COVID-19 travel restrictions) national and international conference presentations (e.g., *American Educational Research Association*). Finally, in partnership with the NSW DoE we will deliver professional learning workshops to support the dissemination of the program in NSW primary schools. Our research team has an impressive track record in the design and dissemination of evidence-based programs in primary and secondary schools (e.g., *iPLAY, and Resistance Training for Teens*).

**REFERENCES**

1. Northouse PG. *Leadership: theory and practice.* Thousand Oaks, CA: : Sage; 2016.

2. Christensen JH, et al. A scoping review of peer-led physical activity interventions involving young people: Theoretical approaches, intervention rationales, and effects. *Youth Soc.* 2020:0044118X20901735.

3. Hulteen RM, et al. Promoting health-enhancing physical activity: A state-of-the-art review of peer-delivered interventions. *Cur Obes Rep.* 2019;8:341-353.

4. Bass BM, et al. *Transformational Leadership.* Mahwah, NJ: Lawrence Erlbaum Associates; 2006.

5. Beauchamp MR, et al. Transformational teaching and physical activity engagement among adolescents. *Exerc Sport Sci Rev.* 2011;39:133-139.

6. Wang G, et al. Transformational leadership and performance across criteria and levels: A meta-analytic review of 25 years of research. *Group Organ Manag.* 2011;36:223-270.

7. Kark R, et al. The two faces of transformational leadership: Empowerment and dependency. *J App Psych.* 2003;88:246-255.

8. Piccolo RF, et al. Transformational leadership and job behaviors: The mediating role of core job characteristics. *Acad Manage J.* 2006;49:327-340.

9. Barling J, et al. Effects of transformational leadership training on attitudinal and financial outcomes: A field experiment. *J App Psych.* 1996;81:827-832.

10. Beauchamp MR, et al. Transformational teaching and adolescent self‐determined motivation, self‐efficacy, and intentions to engage in leisure time physical activity: A randomised controlled pilot trial. *App Psych Health Well.* 2011;3:127-150.

11. Slavich GM, et al. Transformational teaching: Theoretical underpinnings, basic principles, and core methods. *Educ Psych Rev.* 2012;24:569-608.

12. Donnelly J, et al. Physical activity, fitness, cognitive function, and academic achievement in children: A systematic review. *Med Sci Sports Exerc.* 2016;48:1223-1224.

13. Janssen I, et al. Systematic review of the health benefits of physical activity and fitness in school-aged children and youth. *Int J Behav Nutr Phys Act.* 2010;7:doi:10.1186/1479-5868-1187-1140.

14. Schranz N, et al. Results from Australia’s 2018 Report Card on Physical Activity for Children and Youth. *J Phys Act Health.* 2018;15:S315-S317.

15. Borde R, et al. Methodological considerations and impact of school-based interventions on objectively measured physical activity in adolescents: A systematic review and meta-analysis. *Obes Rev.* 2017;18:476–490.

16. Naylor P, et al. Implementation of school based physical activity interventions: A systematic review. *Prev Med.* 2015;72:95-115.

17. Chambers DA, et al. The dynamic sustainability framework: addressing the paradox of sustainment amid ongoing change. *Impl Sci.* 2013;8:117.

18. McCrabb S, et al. Scaling‐up evidence‐based obesity interventions: A systematic review assessing intervention adaptations and effectiveness and quantifying the scale‐up penalty. *Obes Rev.* 2019;20:964-982.

19. Beets M, et al. Identification and evaluation of risk of generalizability biases in pilot versus efficacy/effectiveness trials: a systematic review and meta-analysis. *Int J Behav Nutr Phys Act.* 2020;17:19.

20. Nathan N, et al. Barriers and facilitators to the implementation of physical activity policies in schools: a systematic review. *Prev Med.* 2018;107:45-53.

21. Stapleton P. Teachers are more depressed and anxious than the average Australian. *The Conversation.* 2019. <https://theconversation.com/teachers-are-more-depressed-and-anxious-than-the-average-australian-117267>.

22. Timms C, et al. I just want to teach. *J Educ Admin.* 2007;45:569-586.

23. Yong Z, et al. Causes for burnout among secondary and elementary school teachers and preventive strategies. *Chin Educ Soc.* 2007;40:78-85.

24. Garrick A, et al. Teachers’ priorities for change in Australian schools to support staff well-being. *Asia Pac Educ Res.* 2017;26:117-126.

25. Jenkinson KA, et al. Peer-assisted learning in school physical education, sport and physical activity programmes: a systematic review. *Phys Educ Sport Pedag.* 2014;19:253-277.

26. Yip C, et al. Peer-led nutrition education programs for school-aged youth: a systematic review of the literature. *Health Educ Res.* 2016;31:82-97.

27. MacArthur GJ, et al. Peer‐led interventions to prevent tobacco, alcohol and/or drug use among young people aged 11–21 years: a systematic review and meta‐analysis. *Addict.* 2016;111:391-407.

28. Nathan N, et al. Feasibility and efficacy of the Great Leaders Active StudentS (GLASS) program on children’s physical activity and object control skill competency: a non-randomised trial. *J Sci Med Sport.* 2017;20:1081-1086.

29. Aldrup K, et al. Student misbehavior and teacher well-being: Testing the mediating role of the teacher-student relationship. *Learn Instruc.* 2018;58:126-136.

30. Moher D, et al. CONSORT 2010 explanation and elaboration: updated guidelines for reporting parallel group randomised trials. *BMJ.* 2010;340: doi: 10.1136/bmj.c1869.

31. Hoffmann TC, et al. Better reporting of interventions: template for intervention description and replication (TIDieR) checklist and guide. *BMJ.* 2014;348:g1687.

32. Murray DM. *Design and analysis of group-randomized trials.* Vol 29: Oxford University Press; 1998.

33. Sutherland R, et al. The Physical Activity 4 Everyone cluster randomized trial: 2-Year outcomes of a school physical activity intervention among adolescents. *Am J Prev Med.* 2016;51:195-205.

34. Cohen K, et al. Physical activity and skills intervention: SCORES cluster randomized controlled trial. *Med Sci Sports Exerc.* 2015;47:765-774.

35. Kelloway EK, et al. What we have learned about developing transformational leaders. *Lead Organ Dev J.* 2000.

36. Beauchamp MR, et al. Development and psychometric properties of the transformational teaching questionnaire. *J Health Psych.* 2010;15:1123-1134.

37. Bandura A. Guide for constructing self-efficacy scales. In: Pajares F, Urdan T, eds. *Self-efficacy beliefs of adolescents.* Vol 5. Greenwich, CT: Information Age Publishing; 2006:307-337.

38. McConnell J, et al. Feasibility of an intergenerational-physical-activity leadership intervention. *J Intergen Rel.* 2016;14:220-241.

39. Alberto P, et al. *Applied behavior analysis for teachers.* Australia: Pearson; 2003.

40. Gore J, et al. Effects of professional development on the quality of teaching: Results from a randomized controlled trial of Quality Teaching Rounds. *Teach Teacher Educ.* 2017;68:99-113.

41. Riley N, et al. Findings from the EASY Minds cluster randomized controlled trial: evaluation of a physical activity integration program for mathematics in primary schools. *J Phys Act Health.* 2016;13:198-206.

42. Smith JJ, et al. Smart-phone obesity prevention trial for adolescent boys in low-income communities: The ATLAS RCT. *Pediatr.* 2014;134:e723-e731.

43. Webster EK, et al. Evaluation of the psychometric properties of the Test of Gross Motor Development—third edition. *J Motor Learn Dev.* 2017;5:45-58.

44. Barnett LM, et al. Face validity and reliability of a pictorial instrument for assessing fundamental movement skill perceived competence in young children. *J Sci Med Sport.* 2015;18:98-102.

45. Chandler J, et al. Classification of physical activity intensities using a wrist‐worn accelerometer in 8–12‐year‐old children. *Pediatr Obes.* 2016;11:120-127.

46. Boyle GJ, et al. A structural model of the dimensions of teacher stress. *Br J Educ Psych.* 1995;65:49-67.

47. Collie RJ, et al. Teacher well-being: Exploring its components and a practice-oriented scale. *J Psychoeduc Assess.* 2015;33:744-756.

48. Damschroder LJ, et al. Fostering implementation of health services research findings into practice: a consolidated framework for advancing implementation science. *Implement Sci.* 2009;4:50.

49. Watt HM, et al. Motivations, perceptions, and aspirations concerning teaching as a career for different types of beginning teachers. *Learn Instruct.* 2008;18:408-428.

50. Ding D, et al. The economic burden of physical inactivity: a global analysis of major non-communicable diseases. *The Lancet.* 2016.

51. New South Wales Department of Education. About the school excellence framework. <https://education.nsw.gov.au/teaching-and-learning/school-excellence-and-accountability/sef-evidence-guide/resources/about-sef>. Published 2020. Accessed27th February.
